# Survivin expression in oral squamous cell carcinoma

**DOI:** 10.1038/sj.bjc.6601402

**Published:** 2003-12-09

**Authors:** L Lo Muzio, G Pannone, S Staibano, M D Mignogna, C Rubini, M A Mariggiò, M Procaccini, F Ferrari, G De Rosa, D C Altieri

**Affiliations:** 1 Institute of Dental Sciences, Faculty of Medicine, University of Ancona, Ancona, Italy; 2 Department of Biomorphological and Functional Sciences, Pathology Unit, Faculty of Medicine, University of Naples Federico II, Naples, Italy; 3 Department of Dental Sciences, Faculty of Medicine, University of Naples Federico II, Naples, Italy; 4 Institute of Pathology, University of Ancona, Ancona, Italy; 5 Department of Biomedical Sciences and Human Oncology – Section of General Pathology and Experimental Oncology, University of Bari, Bari, Italy; 6 Department of Cancer Bioilogy and the Cancer Center, University of Massachusetts Medical School, Worcester, MA 01605, USA

**Keywords:** survivin, squamous cell carcinoma, oral mucosa, mouth, apoptosis

## Abstract

A series of 110 cases of oral squamous cell carcinoma (SCC) together with six lymph node and one distant metastatic lesions was analysed for expression of survivin, a recent apoptosis inhibitor, by immunohistochemistry and Western blotting. In total, 91 cases (82.7%) of carcinoma and all metastasis (seven cases, 100%) were positive for survivin expression, with weighted survivin scores ranging from 1 to 4. In contrast, normal oral epithelium did not express survivin. There was no significant correlation between survivin expression and age, sex, tumour size, the presence of lymph node and distant metastases. Survivin expression was increased in poorly differentiated tumours, even if differences were not statistically significant. In contrast, when analysed for prognostic significance, patients with low survivin expression had statistically significant better survival rates than the group with high survivin expression (*P*<0.05). These data suggest that survivin expression may identify cases of oral SCC with more aggressive and invasive phenotype.

The frequency of squamous cell carcinoma (SCC) of the oral mucosa (OSCC) is rapidly increasing. In particular, OSCC constitutes the most frequent malignant tumour of the oral cavity. The incidence of metastasis depends on the degree of cellular differentiation, deep invasion, and site of the primary tumour. However, the clinical behaviour of the single cases of tumours is difficult to predict basing on classical histopathological parameters alone. Biological markers that can identify the lesions with more aggressive phenotype and worse prognosis still need to be identified.

Carcinogenesis is a multistage process involving the activation of oncogenes and the inactivation of tumour suppressor genes. In this context, most human tumours are characterised by an imbalance of regulatory mechanisms controlling cell cycle progression, cell death/viability balance, and apoptosis.

Apoptosis has become a basic tool in developing cancer research and establishing new cancer strategies. Apoptosis, or programmed cell death, is a genetically controlled process, which maintains developmental morphogenesis (Vaux *et al*, 1994), and homeostasis of differentiated organisms by removing senescent, unneeded, or dangerous cells (Sternberg, 1997). Aberrations of this process leading to aberrantly reduced cell death are thought to participate in cancer by promoting increased resistance to therapy and favouring the insurgence of transforming mutations (Jameson, 1998).

Considerable interest has recently focused on the identification of regulators of apoptosis, which may potentially influence the cell death/cell viability balance in cancer. In addition to pro- and antiapoptotic bcl-2 molecules (Reed, 1998), a second gene family of inhibitor of apoptosis (IAP) has been recently identified (Crook *et al*, 1993). Highly evolutionarily conserved from viruses to mammalian cells (Crook *et al*, 1993; Ambrosini *et al*, 1997), IAP proteins target a downstream step in apoptosis by inhibiting the terminal effector caspase-3 and -7 (Deveraux *et al*, 1997; Tamm *et al*, 1998), and by interfering with processing/activation of the pinnacle caspase, caspase-9.

Previous studies demonstrated that expression of antiapoptotic bcl-2 in oral SCC could potentially contribute to tumour progression. This was suggested by the increased expression of bcl-2 protein and mRNA in poorly differentiated oral SCCs as compared with histological normal oral epithelium (Chen *et al*, 2000).

Survivin is a recently characterised IAP protein, which is abundantly expressed in most solid and haematological malignancies, but undetectable in normal adult tissues (Ambrosini *et al*, 1997). Interference with survivin function induces pleiotropic cell-division defects and apoptosis (Li *et al*, 1999), suggesting a potential role at the interface between cell division and apoptosis control. In retrospective trials, survivin expression correlated with unfavourable neuroblastoma (Adida *et al*, 1998a), reduced overall survival in primitive colorectal (Kawasaki *et al*, 1998) and recurrent colorectal cancer (Sarela *et al*, 2000), non-small-cell lung cancer (Monzo *et al*, 1999), breast cancer (Tanaka *et al*, 2000), and increased rates of recurrences in bladder cancer (Swana *et al*, 1999). Consistent with a critical role of apoptosis inhibition in tumour progression, overexpression of survivin in model cancer cell types provided a broad cytoprotective mechanism, counteracting apoptosis induced by FAS/TNF legation, proapoptotic Bax, effector caspases, and various chemotherapeutic drugs (Tamm *et al*, 1998).

In this study, we sought to investigate the potential expression and impact of survivin protein in oral SCC. We found that survivin is expressed in about 80% of oral SCC, and that its degree of expression correlates with a more aggressive phenotype.

## MATERIALS AND METHODS

### Selection of cases

A total of 110 samples from paraffined and 10 from frozen specimens of primary oral SCC, seven from paraffined specimens of lymph node (six cases) and tissue metastases (one case) of OSCCs included in this study were used. Specimens were fixed in 10% neutral-buffered formalin.

None of the patients had been treated previously. They received surgical treatment with curative intention. No case in this study concerned patients with contemporaneous multicentric lesions. Clinical data was reviewed to record sex and age of the patient, and site and size of the lesion. The group consisted of 80 men and 30 women with a mean age of 59.6 years (range 39–78). There were 38 stage I, 29 stage II, 14 stage III, and 29 stage IV. Only 76 patients were analysed for survival rates (the follow-up time was sufficient for statistical analysis, >3 years). Survival was calculated from the date of operation to the date of the latest follow-up visit or death due to cancer. Patients who died of postoperative complications within 30 days were excluded. The histopathological grading was assessed on paraffin haematoxylin–eosin (H&E)-stained sections. Tumour extent was classified according to the TNM system by UICC (UICC, 1987), and tumours were divided into grades 1, 2, and 3 using the WHO classification of histological differentiation.

In all, 10 paraffined and five frozen specimens of healthy oral mucosa were obtained from patients who had undergone routine oral surgical procedures (such as impacted third molars, metaprotesic reactive epithelial hyperplasia, etc.) with the informed consent of the donors. The use of archived human tissues conformed to an informed consent protocol that had been reviewed and approved by the institutional review board of the University of Naples Federico II, Italy.

### Immunohistochemistry

Serial sections (4 *μ*m) from formalin-fixed, paraffin-embedded blocks were cut for each case, and one section stained with H&E was used to confirm the histopathological diagnosis. Only sections containing sufficient epithelium to assess the antibody reactivity with 1000 cells were considered for this study.

Immunohystochemistry was then performed on the remaining sections mounted on poly-L-lysine-coated glass slides. Immunohistochemical staining was carried out with a rabbit polyclonal antibody supplied by NOVUS (NOVUS Biologicals, Littleton, CO, USA) raised against full-length recombinant survivin characterised in previous studies (Grossman *et al*, 1999a; Grossman *et al*, 2001), using the standard streptavidin–biotin–peroxidase complex technique using the Dako LSAB kit (DAKO A/S, Carpinteria, CA, USA) after antigen retrieval by pressure cooking. Briefly, deparaffined sections were immersed in a 10^−3^ M sodium citrate buffer (pH 6.0) after bringing the solution to a boil in a pressure cooker, and then heated two times for 3 min each at a 10-min interval while keeping the pressure indicator valve rising. After quenching in 3% hydrogen peroxide and blocking, the sections were incubated with primary antibody diluted 1 : 50 overnight at 4°C. Biotinylated anti-rabbit immunoglobulin and streptavidin conjugated to horseradish peroxidase were subsequently applied. Finally, 3,3′-diaminobenzidine was used for colour development, and haematoxylin was used for counterstaining. Negative control slides in the absence of primary antibody were included for each staining.

The results of the immunohistochemical staining were evaluated separately by two observers, blind to the histologic diagnosis of the single cases and especially to the follow-up data for the respective patients.

To quantitate the survivin expression, 300 cells were examined in at least five areas at × 400 magnification and a mean percentage of positive tumour cells was determined assigning cases to one of the five following categories: (a) 0, <5%; (b) 1, 5–25%; (c) 2, 26–50%; (d) 3, 51–75%; and (e) 4, >75%. Cases with score of 0 were considered as negative, cases with scores of 1–4 as positive.

### Statistical analysis

Data were analysed using Prism (ver 3.0 for windows) and Stanton A. Glantz (ver 3.0 for dos) softwares. Significant differences (*P*<0.05) between groups were determined using nonparametric Comparisons Test (Kruskall–Wallis test) and Mann–Whitney test and also χ^2^ test. Survival analysis was computed comparing negative (score 0) and low expressing (score 1) with medium (score 2–3) and high (score 4) expressing survivin. Survival curves were analysed according to the method of Kaplan–Meier, and for differences between curves the *P*-value was calculated by the log-rank test. A *P*-value of less than 0.05 was accepted as statistically significant.

### Western blot analysis

Frozen tissues from five samples of oral mucosa and 10 from oral SCCs were homogenised directly into lysis buffer containing 50 mM HEPES, 150 mM NaCl, 1 mM EDTA, 1 mM EGTA, 10% glycerol, 1% Triton-X-100 (1 : 2 weight/volume), 1 mM phenylmethylsulphonyl fluoride (PMSF), 1 *μ*g aprotinin, 0.5 mM sodium orthovanadate, 20 mM sodium pyrophosphate (Sigma Chemical Co, St Louis, MO, USA), and clarified by centrifugation at 14 000 × 10 min. Protein concentrations were estimated using a modified Bradford assay (Bio-Rad, Melville, NY, USA).

In all, 50 *μ*g of total protein extracts was boiled in Laemmli buffer for 5 min before electrophoresis. The samples were subjected to SDS–PAGE (14% polyacrylamide) under reducing conditions. After electrophoresis, proteins were transferred to nitrocellulose membrane (Immobilon Millipore Corporation, Bedford, MA, USA); complete transfer was assessed using prestained protein standards (Bio-Rad, Melville, NY, USA). The membranes were treated for 2 h with blocking solution (5% no fat powdered milk in 25 mM Tris, pH 7.4; 200 mM NaCl; 0.5% Triton X-100, TBS/T), and then the membranes were incubated for 12 h at 4°C with the primary antibody against survivin. After washing with TBS/T and TBS, membranes were incubated with the horseradish peroxidase-conjugated secondary antibody (1 : 5000) for 1 h (at room temperature) and the reaction was detected with enhanced chemiluminescence (ECL) system (Amersham Life Science, UK).

After stripping, membranes were incubated with a monoclonal antibody against beta-actin 1 : 5000 for 1 h at room temperature (Sigma-Aldrich, St. Louis, MO, USA) to ensure an equivalent protein loading in each lane.

## RESULTS

### Survivin expression in normal oral mucosa

Normal oral mucosa specimens showed positivity only in sporadic cells of basal and parabasal layers and were considered negative for survivin expression by immunohistochemistry (score=0), in agreement with previous observations (Lo Muzio *et al*, 2001)

### Survivin expression in oral SCC

**Figure 1:**
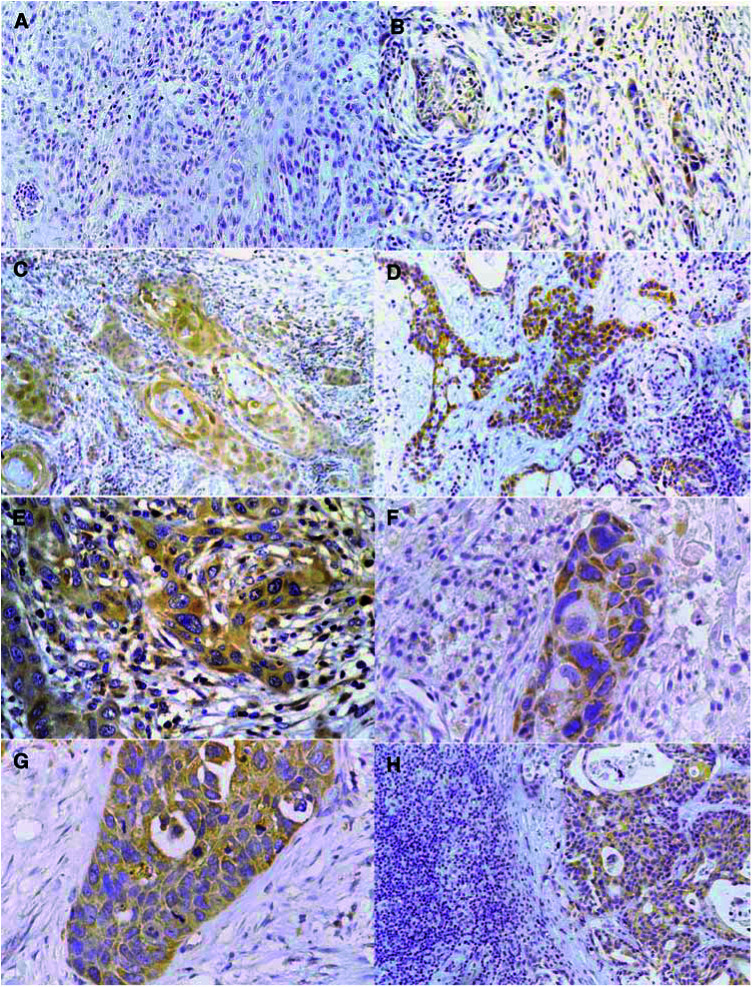
(**A**) A case of moderate-differentiated oral SCC showing no staining for survivin, score 0 (ABC, 150 ×) (**B**) A case of moderate/low-differentiated oral SCC showing staining for survivin, score 1 (ABC, 106 ×) (**C**) A case of moderate differentiated oral SCC showing prevalent cytoplasmatic staining for survivin, score 2 (ABC, 150 ×) (**D**) A case of oral SCC with low grade of differentiation showing cytoplasmatic positivity for survivin, score 4 (ABC, 106 ×) (**E**) A case of oral SCC with low grade of differentiation showing cytoplasmatic positivity for survivin at high power, score 4 (ABC, 250 ×) (**F**) A case of oral SCC with low grade of differentiation showing cytoplasmatic positivity for survivin at high power, score 4 (ABC, 400 ×) (**G**) A case of oral SCC with low grade of differentiation showing cytoplasmatic positivity for survivin at high power, score 4 (ABC, 400 ×) (**H**) A case of lymph node metastasis from oral SCC with definite immunostaining for survivin in tumor cells (ABC, 106 ×).

**Figure 2:**
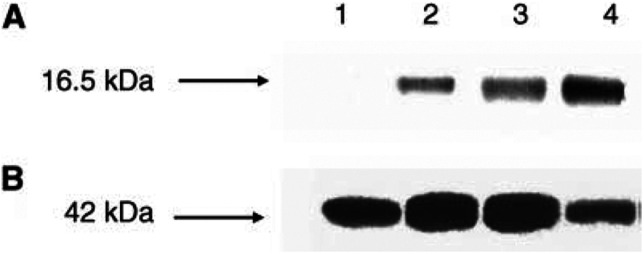
(**A**) Western blot detection of survivin protein in the tissue extracts of normal mucosa (lane 1), well-differentiated SCC (lanes 2), and poorly differentiated SCC (lanes 3 and 4). Proteins (50 *μ*g/lane) was resolved by SDS–PAGE, transferred to nitrocellulose membrane and then incubated with antibody raised against survivin. A specific band was observed sizing about 16.5 kDa by comparison with comigrating size markers (Bio-Rad, Melville, NY, USA). (**B**)The same membrane was stripped and incubated with antibody raised against beta-actin, sizing about 42 kDa (Sigma-Aldrich). The blots are representative of three separate assays.

Immunohistochemical staining for survivin was observed in 20–100% of cancer cells. The immunostaining was prevalently cytoplasmic in poorly differentiated cases with sporadic prominent nuclear staining in well-differentiated areas, as already reported in cervical mucosa (Frost *et al*, 2002). However, in this study, differences in the intracellular pattern of staining that resulted were not statistically significant; for this reason, we recorded the specimens as positive without considering the intracellular localisation of the signal (cytoplasmic or nuclear). In contrast, neighbouring normal tissues did not express survivin. After survivin score, 91 cases (82.7%) of oral mucosa cancers were defined as positive, with weighted survivin scores ranging from 1 to 4 (Figures 1A–D

). Expression of ∼16.5 kDa survivin in tissue extracts of oral SCC was also demonstrated by Western blotting (Figure 2

). All the cases of metastasis enclosed in this study showed survivin positivity.

There was no significant correlation between survivin expression and age, sex, tumour size, or the presence of lymph node and distant metastases (Table 1

**Table 1:** Statistical analysis of survivin score expression and associated clinicopathological findings in oral SCCs

		**Survivin expression**	**Score 0**	**Score 1**	**Score 2**	**Score 3**	**Score 4**				
**Variables**	**No.**	**n. (%)**	**no. (%)**	**no. (%)**	**no. (%)**	**no. (%)**	**no. (%)**	**Mean**	**S.d.**	**S.e.**	**P**
Cases	110	91 (82.7)	19 (17.2)	16 (14.5)	27 (24.5)	24 (21.8)	24 (21.8)				
*Age*											
<60 years	36	30 (83)	6 (5.4)	5 (4.5)	11 (10)	9 (8.1)	5 (4.5)	2.056	1.286	0.214	
>60 years	74	61 (82)	13 (11.8)	11 (10)	16 (14.5)	15 (13.6)	19 (17.2)	2.216	1.436	0.166	0.5310^a^
*Sex*											
Male	80	66 (82)	14 (12.7)	11 (10)	19 (17.2)	18 (16.3)	18 (16.3)	2.188	1.397	0.1562	0.7521^a^
Female	30	25 (83)	5 (4.5)	5 (4.5)	8 (7.2)	6 (5.4)	6 (5.4)	2.100	1.373	0.2507	
*Grading*											
G1	23	18 (78)	5 (5)	2 (1.8)	4 (3.6)	5 (4.5)	7 (6.3)	2.304	1.550	0.3232	
G2	62	51 (82)	11 (10)	12 (10.9)	18 (16.3)	10 (9)	11 (10)	1.968	1.342	0.1705	0.1787^b^
G3	25	22 (88)	3 (2.7)	2 (1.8)	5 (4.5)	9 (8.1)	6 (5.4)	2.520	1.295	0.2590	
*Size (cm)*											
<1.5	36	30 (83)	6 (5.4)	3 (2.7)	11 (10)	8 (7.2)	8 (7.2)	2.250	1.360	0.226	0.6684^a^
>1.5	74	61 (82)	13 (11.8)	13 (11.8)	16 (14.5)	16 (14.5)	16 (14.5)	2.122	1.404	0.163	
*Lymph node metastasis*											
Negative											
Positive (N+)	68	57 (83)	11 (10)	10 (9)	18 (16.3)	14 (12.7)	15 (13.6)	2.176	1.371	0.1662	0.9361^a^
Lymph node metastasis	42	34 (80)	8 (7.2)	6 (5.4)	9 (8.1)	10 (9)	9 (8.1)	2.143	1.424	0.2197	
*Staging*											
I	38	30 (78)	8 (7.2)	4 (3.6)	10 (9)	4 (3.6)	12 (10.9)	2.211	1.527	0.2478	0.2436^b^
II	29	23 (79)	6 (5.4)	4 (3.6)	8 (7.2)	8 (7.2)	3 (2.7)	1.931	1.307	0.2428	
III	14	13 (92)	1 (0.9)	1 (0.9)	2 (1.8)	6 (5.4)	4 (3.6)	2.786	1.188	0.3176	
IV	29	25 (86)	4 (3.6)	7 (6.3)	7 (6.3)	6 (5.4)	5 (4.5)	2.034	1.322	0.2456	

a Mann–Whitney test.

b Nonparametric comparisons test (Kruskall–Wallis test).

). Survivin expression was often increased in poor differentiated tumours, but differences that resulted were not statistically significant. In contrast, when analysed for prognostic significance, patients with low survivin expression had better survival rates than the group with medium and high survivin expression (Figure 3

**Figure 3:**
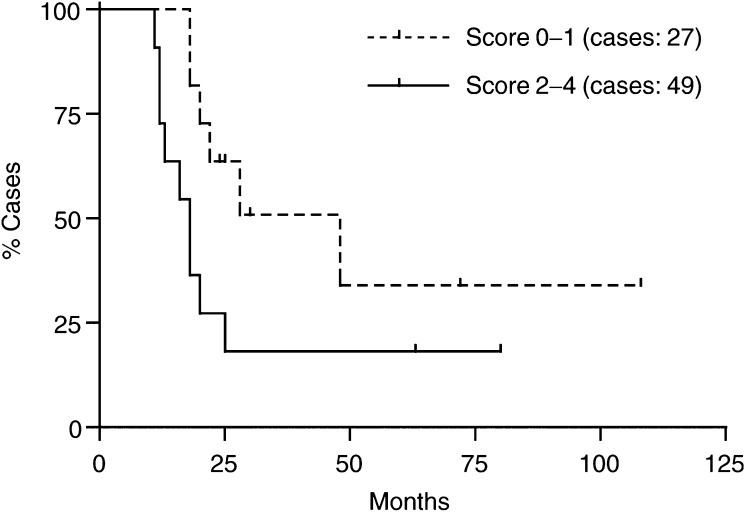
Survival analysis was computed comparing low expressing (score 0–1) with medium/high (score 2–4) expressing survivin. Patients with negative or low score had better survival rates than patients with medium and high score for survivin expression. This difference of survival rates was statistically significant (*P*<0.05).

). This difference of survival rates was statistically significant (*P*<0.05) and the Hazard was 0.2696 with 95% confidence interval ranging from 0.02666 to 0.85475.

## DISCUSSION

In this study, we have shown that a novel antiapoptosis gene, survivin, is prominently expressed in more than 80% of oral SCC. In particular, survivin expression often increased in poorly differentiated tumours, even if differences were not statistically significant. Of more interest, the high survivin expression that resulted correlated with poor survival rates.

Compelling experimental evidence recently suggested that deregulation of apoptosis plays a critical role in the onset and progression of cancer (Thompson, 1995). This conclusion stemmed primarily from the effects of deregulated bcl-2 expression in follicular lymphoma, which resulted in aberrantly prolonged B-cell survival, increased accumulation of transforming mutations, and accelerated transition to more aggressive and therapy-resistant disease (Hua *et al*, 1988). A similar paradigm has also been suggested for other solid malignancies, thus prompting the search for additional molecular markers potentially influencing the cell death/cell viability balance in cancer. In this context, recent studies identified the IAP family protein, survivin, as a novel apoptosis inhibitor selectively overexpressed in most human cancers, but not in normal tissues (Ambrosini *et al*, 1997; Adida *et al*, 1998b). Differently from bcl-2 family molecules, IAP proteins are thought to block a highly evolutionarily conserved step in cell death by binding and inhibiting terminal effector caspases-3 and –7 (Deveraux *et al*, 1998), thus providing a separate and nonredundant pathway of cell viability in cancer.

Here, the presence of survivin in more aggressive and poorly differentiated variants of oral SCC confirms and extends earlier reports of survivin expression in skin cancers (Chiodino *et al*, 1999; Grossman *et al*, 1999b), and suggests its potential predictive/prognostic impact for disease progression. In model cancer cells, expression of the survivin gene was shown to occur exclusively in the G2/M phase in a strict cell cycle-regulated manner (Li *et al*, 1998), thus potentially explaining a preferential expression of survivin in poorly differentiated and metastatic SCC, likely to exhibit high proliferative potential. At a molecular level, survivin localised to mitotic spindle microtubules of dividing cells (Li *et al*, 1998), in a reaction required to preserve apoptosis inhibition. Moreover, *in vitro* targeting experiments using survivin antisense or a dominant negative survivin mutant demonstrated that this unique topography was also required for proper cell division timing and cell progression through mitosis. Consistent with this view, RNA interference of a survivin-homologous BIR1 protein in model organisms also resulted in embryonic lethality due to a profound defect of cytokinesis (Fraser *et al*, 1999). Altogether, these data suggest that survivin expression in aggressive SCC may provide a strong growth advantage factor for tumour progression, affording both protection from broad apoptosis-inducing stimuli/drugs and maintaining proper mitotic progression of the proliferating population. Consistent with this view, recent studies (Glinsky *et al*, 1997) demonstrated that highly metastatic cancers exhibit a higher resistance to apoptotic cell death as compared to low-metastatic counterparts (Glinsky *et al*, 1997).

In summary, these data suggest that survivin expression in oral SCC may identify patients at risk of more aggressive and disseminated disease. This may be relevant for the institution of closer follow-up protocols and/or alternative combined therapeutic regimens. These findings reiterate the importance of deregulation of apoptosis as a critical pathogenetic component of tumour progression, and identify survivin as a potential novel molecular marker of aggressive neoplasia.
